# Supplementary material for: Obesity and Abdominal Obesity in Indian Population: Findings from a Nationally Representative Study of 698,286 Participants
Source: Epidemiologia (Basel). 2023 May 12;4(2):163–72. doi: 10.3390/epidemiologia4020017 (PMC10204471; doi:10.3390/epidemiologia4020017)
Supplement: Supplementary file 1 [file epidemiologia-04-00017-s001.zip › epidemiologia-2313886-supplementary.pdf]

epidemiologia

Table S1. Prevalence of obesity and abdominal obesity by background characteristics among males.

Variable	Obesity	Abdominal obesity
**Age group**		
18-24	4.77	36.46
25-34	10.64	51.87
35-44	13.28	57.98
45-54	13.66	61.59
**Highest Educational Attainment**		
No formal schooling	5.82	49.62
Up to primary	8.20	53.36
Up to secondary	12.32	56.07
College and higher	17.64	57.88
**Marital Status**		
Never married	9.10	54.12
Currently married	11.65	55.80
Others[a]	7.42	49.67
**Household Wealth Status**		
Poorest	3.85	47.09
Poorer	6.45	51.72
Middle	9.82	54.18
Richer	14.71	58.00
Richest	21.31	61.32
**Zone of Residence**		
North	12.52	57.94
North-East	7.61	46.39
Central	8.93	54.67
East	7.30	58.40
West	12.06	46.71
South	18.26	56.54
**Place of Residence**		
Urban	16.76	58.39
Rural	9.04	53.04
**Current Smoker/Tobacco use**		
No	14.91	56.72
Yes	7.93	52.85
**Current Alcohol Consumption**		
No	12.11	54.97
Yes	10.04	54.78

[a] Divorced/Widowed/Separated/Don't Know.

Table S2. Prevalence of obesity and abdominal obesity by background characteristics among females.

Variable	Obesity	Abdominal obesity
**Age group**		
18-24	4.75	50.32
25-34	12.65	57.04
35-44	19.42	61.77
45-54	20.68	66.14

**Highest Educational Attainment**		
No formal schooling	10.30	59.43
Up to primary	15.54	60.12
Up to secondary	20.74	61.98
College and higher	23.99	64.94
**Marital Status**		
Never married	12.00	61.22
Currently married	16.73	61.34
Others[a]	15.06	58.94
**Household Wealth Status**		
Poorest	5.38	59.46
Poorer	9.64	58.88
Middle	15.17	59.44
Richer	21.06	61.34
Richest	27.98	64.76
**Zone of Residence**		
North	18.70	69.89
North-East	8.87	69.33
Central	14.15	58.41
East	11.39	70.74
West	15.00	47.27
South	24.08	55.52
**Place of Residence**		
Urban	24.01	64.13
Rural	12.57	59.25
**Current Smoker/Tobacco use**		
No	17.13	61.18
Yes	8.66	57.77
**Current Alcohol Consumption**		
No	16.43	60.83
Yes	7.87	61.88

[a] Divorced/Widowed/Separated/Don't Know.

Table S3. Crude and adjusted odds ratio (95% CI) estimates of the factors associated with obesity and abdominal obesity among males.

**Variable**	**Obesity**		**Abdominal Obesity**	
	**COR (95% CI)**	**AOR (95% CI)**	**COR (95% CI)**	**AOR (95% CI)**
**Age group (In years)**				
18-24	Ref	Ref	Ref	Ref
25-34	2.38*** (2.20-2.59)	2.00*** (1.82-2.20)	1.95*** (1.86-2.03)	1.70*** (1.61-1.79)
35-44	3.26*** (3.00-3.53)	2.67*** (2.40-2.96)	2.77*** (2.65-2.90)	2.34*** (2.21-2.49)
45-54	3.41*** (3.14-3.71)	2.85*** (2.56-3.18)	3.18*** (3.03-3.33)	2.72*** (2.55-2.89)
**Highest Educational Attainment**				
No formal schooling	Ref	Ref	Ref	Ref
Up to primary	1.30*** (1.16-1.46)	1.23*** (1.10-1.38)	1.07* (1.00-1.14)	1.14*** (1.07-1.21)

Up to secondary	1.74*** (1.59-1.91)	1.52*** (1.38-1.67)	1.00 (0.95-1.06)	1.21*** (1.15-1.28)
College and higher	2.17*** (1.96-2.40)	1.61*** (1.44-1.79)	0.97 (0.92-1.03)	1.21*** (1.13-1.29)
**Marital Status**				
Never married	Ref	Ref	Ref	Ref
Currently married	2.25*** (2.12-2.40)	1.47*** (1.35-1.59)	2.22*** (2.15-2.30)	1.36*** (1.29-1.43)
Others[a]	1.57*** (1.29-1.91)	1.21 (0.99-1.49)	1.59*** (1.41-1.79)	1.00 (0.88-1.14)
**Household Wealth Status**				
Poorest	Ref	Ref	Ref	Ref
Poorer	1.86*** (1.68-2.07)	1.65*** (1.49-1.84)	1.25*** (1.19-1.31)	1.27*** (1.21-1.34)
Middle	2.90*** (2.62-3.20)	2.32*** (2.08-2.58)	1.44*** (1.37-1.52)	1.45*** (1.37-1.54)
Richer	4.36*** (3.94-4.82)	3.25*** (2.91-3.62)	1.75*** (1.65-1.85)	1.72*** (1.61-1.83)
Richest	6.98*** (6.31-7.72)	4.98*** (4.43-5.60)	2.12*** (1.99-2.25)	1.97*** (1.83-2.13)
**Zone of Residence**				
North	Ref	Ref	Ref	Ref
North-East	0.70*** (0.63-0.78)	1.22*** (1.09-1.35)	0.47*** (0.43-0.51)	0.54*** (0.50-0.60)
Central	0.58*** (0.53-0.64)	0.88** (0.80-0.96)	0.71*** (0.66-0.77)	0.85*** (0.78-0.92)
East	0.52*** (0.47-0.58)	0.95 (0.85-1.06)	0.84*** (0.77-0.92)	1.07 (0.97-1.17)
West	0.78*** (0.70-0.88)	0.89* (0.80-0.99)	0.47*** (0.42-0.52)	0.47*** (0.42-0.52)
South	1.55*** (1.41-1.70)	1.61*** (1.47-1.76)	0.75*** (0.68-0.81)	0.70*** (0.64-0.77)
**Place of Residence**				
Urban	Ref	Ref	Ref	Ref
Rural	0.48*** (0.45-0.51)	0.86*** (0.81-0.92)	0.75*** (0.71-0.79)	0.92*** (0.86-0.98)
**Current Smoker/Tobacco use**				
No	Ref	Ref	Ref	Ref
Yes	0.75*** (0.71-0.79)	0.80*** (0.75-0.84)	1.12*** (1.09-1.16)	0.93*** (0.89-0.96)
**Current Alcohol Consumption**				
No	Ref	Ref	Ref	Ref
Yes	1.00 (0.94-1.06)	1.03 (0.97-1.10)	1.16*** (1.12-1.20)	1.01 (0.97-1.06)

*: *p<0.05*; **: *p<0.01*; ***: *p<0.001*; AOR: Adjusted Odds Ratio; CI: Confidence Interval; COR: Crude Odds Ratio. [a] Divorced/Widowed/Separated/Don't Know.

Table S4. Crude and adjusted odds ratio (95% CI) estimates of the factors associated with obesity and abdominal obesity among females.

**Variable**	**Obesity**		**Abdominal Obesity**	
	**COR (95% CI)**	**AOR (95% CI)**	**COR (95% CI)**	**AOR (95% CI)**

**Age group (In years)**				
18-24	Ref	Ref	Ref	Ref
25-34	3.06*** (2.97-3.16)	2.31*** (2.23-2.39)	1.46*** (1.44-1.48)	1.30*** (1.28-1.32)
35-44	5.29*** (5.14-5.45)	3.99*** (3.85-4.13)	1.88*** (1.85-1.91)	1.67*** (1.63-1.70)
45-54	5.89*** (5.70-6.08)	4.50*** (4.33-4.68)	2.30*** (2.26-2.35)	2.06*** (2.01-2.11)
**Highest Educational Attainment**				
No formal schooling	Ref	Ref	Ref	Ref
Up to primary	1.18*** (1.15-1.22)	1.22*** (1.19-1.26)	0.92*** (0.90-0.94)	1.00 (0.98-1.02)
Up to secondary	1.11*** (1.09-1.14)	1.30*** (1.26-1.33)	0.80*** (0.79-0.82)	1.02* (1.00-1.04)
College and higher	0.90*** (0.87-0.93)	1.17*** (1.13-1.21)	0.71*** (0.69-0.72)	1.02 (1.00-1.05)
**Marital Status**				
Never married	Ref	Ref	Ref	Ref
Currently married	4.16*** (4.03-4.30)	1.95*** (1.88-2.03)	1.75*** (1.73-1.78)	1.31*** (1.28-1.34)
Others[a]	4.07*** (3.88-4.27)	1.75*** (1.66-1.85)	1.87*** (1.81-1.93)	1.27*** (1.23-1.31)
**Household Wealth Status**				
Poorest	Ref	Ref	Ref	Ref
Poorer	1.92*** (1.85-2.00)	1.78*** (1.71-1.85)	1.06*** (1.04-1.08)	1.08*** (1.06-1.10)
Middle	3.08*** (2.97-3.19)	2.62*** (2.52-2.72)	1.12*** (1.10-1.15)	1.16*** (1.14-1.19)
Richer	4.57*** (4.41-4.73)	3.66*** (3.52-3.81)	1.21*** (1.18-1.23)	1.25*** (1.22-1.28)
Richest	6.87*** (6.62-7.13)	5.21*** (4.99-5.43)	1.33*** (1.29-1.36)	1.33*** (1.29-1.37)
**Zone of Residence**				
North	Ref	Ref	Ref	Ref
North-East	0.46*** (0.44-0.48)	0.81*** (0.78-0.85)	0.78*** (0.74-0.82)	0.88*** (0.83-0.92)
Central	0.58*** (0.56-0.60)	0.91 *** (0.87-0.94)	0.42*** (0.40-0.44)	0.46*** (0.44-0.48)
East	0.47*** (0.45-0.49)	0.90*** (0.86-0.94)	0.71*** (0.67-0.74)	0.80 ***(0.76-0.84)
West	0.72*** (0.69-0.76)	0.77*** (0.73-0.80)	0.25*** (0.24-0.26)	0.24*** (0.23-0.26)
South	1.43*** (1.37-1.49)	1.45*** (1.39-1.50)	0.39*** (0.37-0.41)	0.37*** (0.35-0.39)
**Place of Residence**				
Urban	Ref	Ref	Ref	Ref
Rural	0.39*** (0.38-0.40)	0.72*** (0.70-0.74)	0.82*** (0.80-0.85)	0.85*** (0.82-0.88)
**Current Smoker/Tobacco use**				
No	Ref	Ref	Ref	Ref

Yes	0.80*** (0.78-0.83)	0.83*** (0.80-0.86)	1.08*** (1.05-1.10)	0.90*** (0.88-0.92)
**Current Alcohol Consumption**				
No	Ref	Ref	Ref	Ref
Yes	0.84*** (0.78-0.89)	0.98 (0.92-1.05)	1.22*** (1.17-1.27)	1.09*** (1.04-1.14)

**: p<0.05; **: p<0.01; ***: p<0.001;* AOR: Adjusted Odds Ratio; CI: Confidence Interval; COR: Crude Odds Ratio; [a] Divorced/Widowed/Separated/Don't Know.
